# Next-generation epidemiology: the role of high-resolution molecular phenotyping in diabetes research

**DOI:** 10.1007/s00125-020-05246-w

**Published:** 2020-08-25

**Authors:** Paul W. Franks, Hugo Pomares-Millan

**Affiliations:** 1 Department of Clinical Sciences, Genetic and Molecular Epidemiology Unit, Clinical Research Centre, Lund University, Jan Waldenströmsgata 35, Skåne University Hospital, SE-20502 Malmö, Sweden; 2 Harvard T.H. Chan School of Public Health, Boston, MA USA

**Keywords:** Bioinformatics, Biomarkers, Diabetes, Epidemiology, Genetics, Omics, Review

## Abstract

Epidemiologists have for many decades reported on the patterns and distributions of diabetes within and between populations and have helped to elucidate the aetiology of the disease. This has helped raise awareness of the tremendous burden the disease places on individuals and societies; it has also identified key risk factors that have become the focus of diabetes prevention trials and helped shape public health recommendations. Recent developments in affordable high-throughput genetic and molecular phenotyping technologies have driven the emergence of a new type of epidemiology with a more mechanistic focus than ever before. Studies employing these technologies have identified gene variants or causal loci, and linked these to other omics data that help define the molecular processes mediating the effects of genetic variation in the expression of clinical phenotypes. The scale of these epidemiological studies is rapidly growing; a trend that is set to continue as the public and private sectors invest heavily in omics data generation. Many are banking on this massive volume of diverse molecular data for breakthroughs in drug discovery and predicting sensitivity to risk factors, response to therapies and susceptibility to diabetes complications, as well as the development of disease-monitoring tools and surrogate outcomes. To realise these possibilities, it is essential that omics technologies are applied to well-designed epidemiological studies and that the emerging data are carefully analysed and interpreted. One might view this as next-generation epidemiology, where complex high-dimensionality data analysis approaches will need to be blended with many of the core principles of epidemiological research. In this article, we review the literature on omics in diabetes epidemiology and discuss how this field is evolving.

**Electronic supplementary material:**

The online version of this article (10.1007/s00125-020-05246-w) contains a slide of the figure for download, which is available to authorised users.

**Figure:**
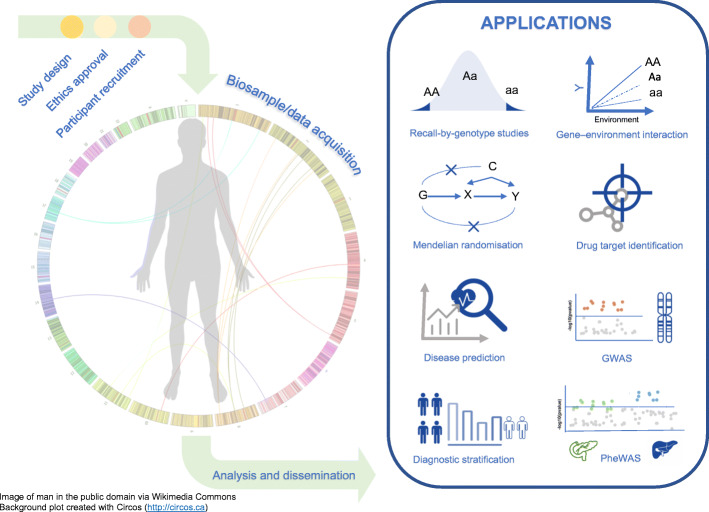
Graphical abstract

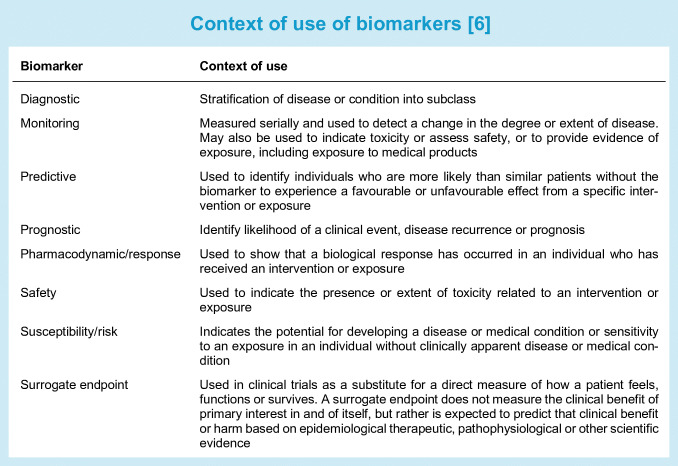

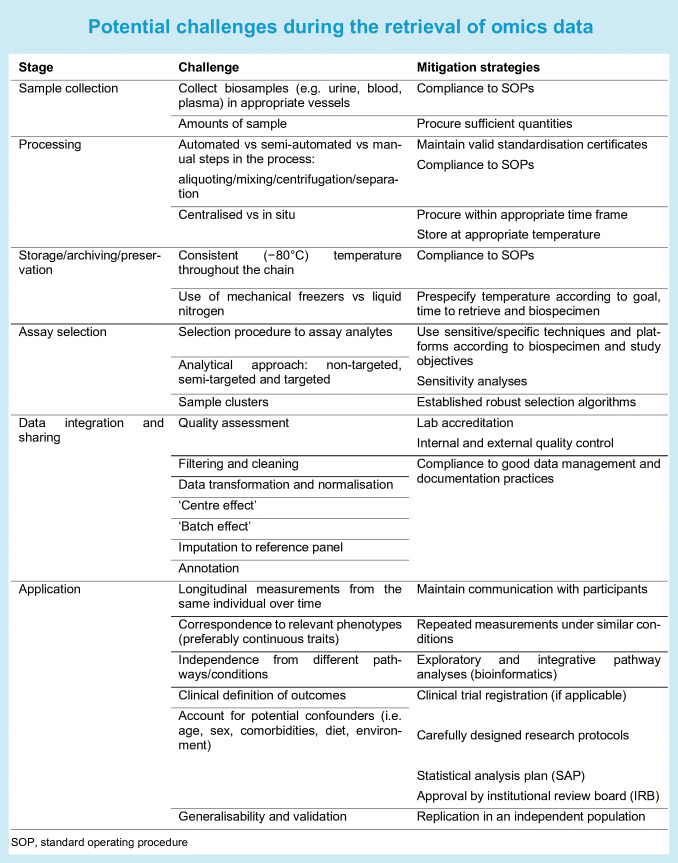

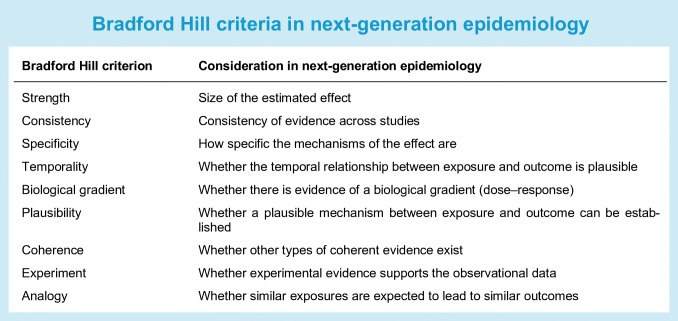







## Introduction

The aetiology and clinical presentation of diabetes often differ greatly from one patient to the next, as do patients’ responses to therapies and the rates at which they develop complications. Identifying biomarkers that aid the prediction and prevention of diabetes by helping stratify populations depending on (1) sensitivity to risk factors, (2) likely response to therapies, and (3) susceptibility to diabetes complications, as well as identifying biomarkers for disease monitoring and as surrogate outcomes, are major priorities in diabetes research.

Biomarkers are also used extensively in diabetes epidemiology as intermediate exposure or outcome variables when seeking to understand disease aetiology. For example, HbA_1c_ and blood glucose concentrations are the principal biomarkers of diabetes, and measures of blood concentrations of insulin, proinsulin, lipids, inflammatory cytokines and adipokines are often used to study the determinants or consequences of diabetes.

The development of high-throughput molecular genotyping and phenotyping assays has led to a new field of omics research, which has seen the discovery of many types of biological variants influencing diabetes. This review explores diabetes epidemiology, with specific focus on omics research. How the next generation of epidemiological studies and methods are likely to evolve and contribute to understanding diabetes is also discussed.

### What are biomarkers?

The term ‘biomarker’ was first used in the field of petroleum chemistry in the late 1960s [1], appearing a few years later in the biomedical literature to describe the role of serum RNase as an indicator of renal function [2]. The National Institutes of Health’s Biomarkers Definitions Working Group subsequently defined a biomarker as ‘A characteristic that is objectively measured and evaluated as an indicator of normal biological processes, pathogenic processes, or pharmacologic responses to a therapeutic intervention’ [3]; while other definitions have followed, this early one remains widely used. Biomarkers have multiple uses in biomedicine, including in drug trials, though discussion of their use in such trials is outside the remit of this article. Nevertheless, the US Food and Drug Administration’s (FDA) ‘context of use’ framework for the use of biomarkers in drug trials provides a reasonable foundation upon which biomarkers in many areas of epidemiology research can be considered. Briefly, the FDA cites seven specific contexts within which biomarkers can be used in drug trials: (1) diagnosis (for patient selection), (2) monitoring (disease development, toxicity, exposure), (3) prediction (effects of interventions or exposures), (4) prognosis (patient stratification and/or enrichment), (5) pharmacodynamics/response (efficacy: surrogate endpoints and/or biological response to treatment), (6) safety, and (7) susceptibility/risk (potential to develop disease or exposure sensitivity) (see www.fda.gov/drugs/cder-biomarker-qualification-program/context-use) [4, 5]. An extended overview is provided in the Text box ‘Context of use of biomarkers’ [6].

## The evolution of omics in epidemiology

Comprehensive molecular phenotyping in very large cohort collections has facilitated the discovery of many previously unknown biological pathways, providing substrates for drug development pipelines, the optimisation of non-pharmacological interventions, disease-monitoring technologies and disease-prediction algorithms. This has involved genome-wide association studies (GWAS) and their statistical aggregation through meta-analysis [7].

GWAS usually require large cohort collections to afford adequate power, mainly because many parallel hypotheses are tested (>1 million) and risk of type 1 error (false-positive discovery) is consequently high. A limitation of GWAS is the inability to detect certain types of variants, either because they were absent within the populations used to inform the content of GWAS arrays or imputation panels, or because the array is simply not designed to detect certain types of variant. This can prove problematic when studying rare variants [8], but also applies to non-SNP variants such as insertion–deletion polymorphisms (indels) [9, 10], although this may be less of a concern than initially thought [8]. Alternatively, whole-genome sequencing involves the interrogation of each accessible base pair in the nuclear genome in a manner that is largely agnostic to the identity of the specific variants. Thus, with sequencing, previously unknown variants (or at least those not included in genotyping arrays) can be discovered and related to phenotypic variation. As an example, homozygote carriers of the *TBC1D4* nonsense p.Arg684ter allele, common in the Greenland Inuit population but rare elsewhere, have ~10-fold increased odds of type 2 diabetes [11]. This causal variant is tagged by genotypes captured in certain arrays, but the causal variant itself is not captured; thus, its detection required de novo exome sequencing of DNA from Inuit trios (mother, father and a child).

**Fig. 1:**
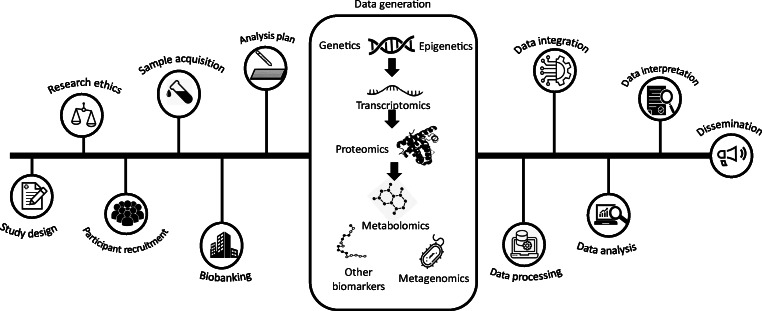
Omics studies workflow. Initial stages of omic studies involve the ethical approval of the study protocol (research ethics) and written consent (participant recruitment) provided by the participants where biological samples are drawn for further analyses. Downstream stages include critical steps, i.e. sample storage and processing, data generation, and data analysis (integration, interpretation and dissemination). This figure is available as a downloadable slide

Many other types of omics data (e.g. transcriptomics, proteomics, microRNAs, epigenetics, peptidomics, metabolomics, lipidomics, metagenomics) can also be derived from stored biosamples using targeted assays, arrays or sequencing technologies, depending on storage procedures [12] (see Table 1, and Fig. 1 and Text box: Potential challenges during the retrieval of omics data).

**Table 1:** Overview of omics technologies

Technology	Term coined by, year	Concept	Objective	Platform(s)	Reference
Genomics	Thomas H. Roderick, 1986	Genes, their mapping and functions	Identify genetic functionality	Next-generation sequencing; arrays; bioinformatics	[51]
Genetics	William Bateson, 1905	Genes and their variations	Identify genetic makeup, heredity and functionality	Next-generation sequencing; arrays; bioinformatics	[52]
Metagenomics	Jo Handelsman, 1998	Analysis of the interacting population of organisms in the body	Identify genetic functionality from environmental sources (e.g. gut, oral microbiome)	Microbial genome sequencing (16S rRNA/“Shotgun”); bioinformatics	[53]
Nutrigenomics	Nancy Fogg-Johnson and Alex Merolli, 1996	The relationship between nutritional physiology and genetic makeup	Measure dietary effects on the transcriptome or metabolome	RNA-Seq; Microarray; Chromatography; MS; NMR	[54]
Proteomics	Marc Wilkins, 1995	Proteins	Identify structure and activity of proteins expressed	MS; protein arrays	[55]
Metabolomics/ Metabonomics	Steven Oliver, 1998 /Jeremy Nicholson, 1999	Metabolites	Identify and quantify molecules associated with physiological and pathological effects	Chromatography; MS; NMR	[55, 56]
Epigenetics	Conrad Waddington, 1940	DNA methylation and histone modifications	Study processes that regulate how and when certain genes are turned on and turned off	Next-generation sequencing; arrays; bioinformatics	[57]
Epigenomics	NA, 2006	DNA methylation, chromatin and histone modifications in the genome	Analyse epigenetic changes across many genes in a cell or entire organism	Next-generation sequencing; RNA-Seq; arrays; bioinformatics; ChIP-Seq; ATAC-Seq	[58]
Glycomics	Raymond Dwek, 1982	Cellular carbohydrates	Identify and quantify glycomic molecules	Chromatography; MS; NMR	[59]
Lipidomics	NA, 2003	Cellular lipids	Identify and quantify lipids	Chromatography; MS; NMR	[60]
Transcriptomics	Charles Auffray, 1996	mRNA	Identify genetic transcription and activity intensity	RNA-Seq; arrays	[61]

ATAC-Seq, assay for transposase-accessible chromatin using sequencing; ChIP-Seq analysis, chromatin immunoprecipitation followed by sequencing; NA, not attributed; RNA-Seq, RNA sequencing

## Epidemiology and its role in diabetes research

Epidemiology, the study of disease, its risk factors and its consequences within human populations, has been a cardinal feature of diabetes research for almost a century. In the Whitehall II Study, for example, 6538 British civil servants, initially free from diabetes, were studied repeatedly for about a decade [13]. An analysis of these data showed that over the decade preceding type 2 diabetes diagnosis, fasting and post-load blood glucose concentrations gradually increased, deteriorating sharply in the final 3 years. Compensatory changes in estimated insulin production and insulin sensitivity also occurred, whereby insulin sensitivity declined rapidly during the final 5 years before diagnosis and insulin production initially increased from years four to three pre-diagnosis, only to decline rapidly thereafter. In those who did not develop diabetes, fasting blood glucose concentrations and insulin production remained materially unchanged throughout follow-up, whereas post-load glucose rose gradually, and insulin sensitivity declined throughout follow-up at rates similar to those seen in participants who developed diabetes.

‘Correlation’ does not necessarily mean ‘causation’; some types of epidemiological analyses, such as those focused on prediction (for example, of risk of developing diabetes, of susceptibility to risk factors or of treatment success/failure) do not always require that the relationships between exposures and outcomes are causal for the results to be clinically useful. Similarly, descriptive epidemiology does not seek to establish cause and effect, instead focusing on detailing the patterns and distributions of disease. However, in aetiological epidemiology, where attention is often placed on understanding mechanisms of action, establishing causality is paramount, especially where focus is on discovery of novel drug targets that perturb pathways influencing diabetes or diabetes complications.

The major barriers to causal inference in epidemiology are chance, bias and confounding. These challenges can be addressed to some extent by applying certain data analysis conventions, such as the Bradford Hill criteria [14] (see Text box: Bradford Hill criteria in next-generation epidemiology). Of the many quantitative approaches for causal inference, Mendelian randomisation (MR), which often utilises genetic variants as the ‘causal instrument’, is popular. SNPs, unlike most other types of biomarker, remain constant throughout life. Thus, unlike most other biomarkers, there is no need to reassess genotypes once on file. This stability also means that cross-sectional associations between genotypes and traits can be considered unidirectional*.* MR exploits these strengths as well as the random assortment of genotypes to minimise the impact of confounding and reverse causality [15].

Several branched-chain amino acids (BCAAs) such as isoleucine, leucine and valine have been among the ~100 biomarkers reproducibly associated with type 2 diabetes incidence in large observational studies [16]. Of these, alanine aminotransferase, proinsulin and uric acid are also supported by causal evidence from MR studies [17]. Early studies exploring the causal link between vitamin D and diabetes, using a genetic instrument comprised of variants associated with circulating levels, showed conflicting evidence [18–20]. However, in later studies, when the sample size and the instrumental variables were expanded and the genetic instrument included variants regulating the synthesis, transport and catabolism of vitamin D, a causal relationship was evident [21].

Most MR studies have focused on prevalent diabetes, with relatively few (about ten) biomarkers being causally associated with incident type 2 diabetes [22]. A recent elegant analysis [23] of biomarkers in incident diabetes reported that 35 biomarkers have been studied in cohorts totalling at least 1000 individuals with type 2 diabetes, only one of which (ferritin) yielded strong observational and MR evidence to support a causal role in diabetes incidence. In general, the biomarkers examined did not enhance the accuracy of type 2 diabetes prediction models, and those that did were generally markers of glycaemic control.

Although MR is often viewed as a highly robust means of inferring causal relationships, the approach has noteworthy caveats [24]. For example, Haworth and colleagues [25] describe geographically aligned genetic structures associated with traits such educational attainment, BMI and number of siblings, using data from the UK Biobank, which raises concerns about the validity of some published MR analyses.

## The value of biobanked samples and longitudinal cohorts

Biorepositories have existed for several decades, although the term ‘biobank’ was first used in the late 1990s [26]. The manner in which biobanks would be used today could not have been known when they were first initiated. Nevertheless, modern genotyping and phenotyping technologies have helped raise the value of many biobanks that were initiated long before these technologies were invented. In the UK, the Department of Health, the Medical Research Council, the Scottish Executive, and the Wellcome Trust invested UK£62m to establish UK Biobank, a prospective cohort study of 500,000 adults from the UK. Roughly 5% of the cohort has prevalent or incident diabetes [27], representing a large case group that is set to expand as the cohort ages. Established as a non-profit project in the early 2000s, UK Biobank has proved to be an outstanding resource for aetiological epidemiology owing to the extensive genotyping, relatively deep phenotyping and linkage with electronic health records. The thousand or so papers published in the past 7 years using UK Biobank data have spanned many health topics, with several dozen papers relating explicitly to diabetes. A common criticism of biobank research is that many are too small to stand alone and have thus formed parts of larger biobank networks, where data harmonisation has been challenging owing to the variety of methods deployed to assess the same underlying exposures and outcomes. Thus, as a single, large, standardised bioresource, UK Biobank has helped to address this criticism.

## Next-generation epidemiology

The idea of genotyping and repeatedly phenotyping the same individual using multiple omics platforms was stimulated by a study in a single adult man who underwent deep omics profiling (genomics, transcriptomics, proteomics, metabolomics and autoantibodies) once daily for 14 months [28]. This analysis provided evidence that by integrating dense personal omics data, temporal patterns could be identified to predict subsequent shifts in health and disease markers. While the ‘*n* of 1’ nature of this study limits its generalisability, the technical approaches deployed inspired others to undertake epidemiological studies involving deep phenotyping of existing biosamples, as well as new studies where participants were repeatedly assessed using digital and serological assays to profile temporal patterns related to the development or progression of diabetes.

Applying modern molecular phenotyping technologies to samples stored from historical cohorts is highly pragmatic, particularly when the cohort has a long follow-up and clinical events have accrued. The European Prospective Investigation into Cancer and Nutrition (EPIC)-InterAct (*n* = 12,500 incident cases and *n* = 16,000 reference cohort) is one of the largest nested case–cohort studies of incident diabetes. The study comprised subgroups of participants identified from a larger European prospective cohort study (EPIC, *N* = 500,000). The aim of InterAct was to assess gene–lifestyle interactions, but it has subsequently been used to address many other questions, including those focused on the role of diet in diabetes. Among the biomarkers analysed were plasma phospholipid fatty acids by gas chromatography. Imamura et al [29] used these data to derive a dietary fatty acid score, which they found to be inversely related to incident diabetes. In post hoc analyses, the same score was inversely associated with higher levels of liver enzymes, inflammatory markers, fasting glucose, triacylglycerols and adiposity. Genetic analyses were performed to determine whether these findings might be confounded by obesity or insulin resistance, which they were not.

UK Biobank has addressed some of the limitations of older cohorts by undertaking deep phenotyping at an unprecedented scale, with MRI scans being performed to determine tissue composition, serological samples collected for GWAS, metabolomic and telomere analyses, and validated health outcomes obtained through record linkage. A recent public–private partnership contributed a further UK£200m to undertake whole-genome sequencing of 500,000 UK Biobank participants and pilot work is underway to explore the use of proteomics assays.

In Europe, the Innovative Medicines Initiative (IMI), a partnership between the European Commission, top academic institutions, the European Federation of Pharmaceutical Industries and Associations (EFPIA) and other partners, has invested more than €230m in projects seeking to discover biomarkers that might lead to novel diabetes drug targets, enhance monitoring and/or aid the design of diabetes drug trials. Of these, one IMI consortium (Diabetes Research on Patient Stratification [DIRECT]) established new prospective cohort studies enrolling ~3000 participants at risk of or with newly diagnosed diabetes [30, 31]. The project’s primary objective was to discover biomarkers for glycaemic deterioration before and after the onset of type 2 diabetes and included extensive deep phenotyping at baseline and throughout follow-up. Several other IMI diabetes projects have relied predominantly on assimilating, assaying and mining data from existing epidemiological cohorts for the discovery of diabetes-relevant biomarkers (see Table 2). In the US, the Accelerating Medicines Partnership has genotyped DNA from multiple diabetes case−control studies and assimilated these and other genetic and phenotypic summary data to provide public-access genomics resources (e.g. www.type2diabetesgenetics.org) [32]. In Finland, the FinnGen project [33] has brought together universities, hospitals, biobanks and pharmaceutical companies to study the genetic bases of common and rare diseases, with a focus on biomedical innovation and drug development. In Sweden, academic institutions, hospitals, government and charitable trusts have partnered to research and deliver genomic medicines through Genome Medicine Sweden (https://genomicmedicine.se/en/) [34]. Elsewhere, the UK Government established a company (Genomics England) to deliver genomics medicine to the population of England, with several highly ambitious projects, including the 100,000 Genomes project [35], which is primarily focused on cancers and rare diseases, but which will also include ~8000 families with rare inherited metabolic and endocrine diseases.

**Table 2:** Examples of IMI public–private initiatives in diabetes

Acronym/Study name	Objective	Diabetes context	Total cost (€)	Ref.	Status	Website
IMI-SUMMIT: Surrogate markers for micro- and macrovascular hard endpoints for innovative diabetes tools	Assess biomarkers for diabetes complications	Diabetic complications in T2D	34,812,081	[62]	Final report	www.imi-summit.eu/
IMI-RHAPSODY: Risk Assessment and Progression of Diabetes	Assess glycaemic deterioration before and after the onset of type 2 diabetes	Prediabetes/T2D	18,488,749	-	Ongoing	https://imi-rhapsody.eu/
IMI-INNODIA: Translational Approaches to Disease Modifying Therapy of Type 1 Diabetes: An Innovative Approach Towards Understanding and Arresting Type 1 Diabetes	Advance the understanding of type 1 diabetes	T1D	36,563,723	-	Ongoing	www.innodia.eu/
IMI-BEAT-DKD: Biomarker Enterprise to Attack DKD	Assess diabetic kidney disease	Diabetic complications in T2D	30,163,037	[63]	Ongoing	www.beat-dkd.eu/
IMI-CARDIATEAM^a^: Cardiomyopathy in Type 2 Diabetes Mellitus	Assess diabetic cardiomyopathy	T2D	12,882,500	–	Ongoing	https://cardiateam.eu/
IMI-Hypo-RESOLVE: Hypoglycaemia – Redefining Solutions for Better Lives	Assess diabetic hypoglycaemia	Diabetic complications in T1D	26,774,583	–	Ongoing	https://hypo-resolve.eu/
IMI-DIRECT^a^: Diabetes Research on Patient Stratification	Identify diabetes subtypes and determine the most appropriate treatments	Prediabetes/T2D	46,484,127	[64]	Final report	www.direct-diabetes.org/

^a^Involves new cohort generation

DKD, diabetic ketoacidosis; IMI, Innovative Medicines Initiative; T1D, type 1 diabetes; T2D, type 2 diabetes

Epidemiological cohorts are sometimes used to provide sampling frames from which participants with specific phenotypic or genetic characteristics are recalled for experimental studies or complex in vivo measurements. Recall-by-genotype studies are especially appealing, as the feature upon which participants are recalled (genotype) is not subject to change and this paradigm can be much more powerful for the assessment of gene–treatment interactions than conventional trials [36]. METSIM is a prospective cohort study of Finnish men. In a recent recall-by-genotype study (*n* = 45) nested within METSIM [37], p.Pro50Thr AKT2 variant carriers and common allele homozygous controls were recalled to investigate the effects of the p.Pro50Thr AKT2 variant on insulin-stimulated glucose uptake. In this study, carriers of the risk allele showed reductions in glucose uptake (39.4%) and rate of endogenous glucose production (55.6%) after insulin stimulation compared with non-carriers. Glucose uptake was reduced primarily in musculoskeletal tissue.

## Analytical challenges and emerging solutions

The analysis of dense multiomics datasets has proven formidable. To address some of the computational challenges, machine learning methods have been applied to determine hidden structures that are informative of disease aetiology or prognosis [38, 39]. Emphasis has been placed on the reclassification of the diagnosis of type 2 diabetes into subtypes. The principle of subclassifying diabetes using genetics and applying this knowledge to guide therapeutic decision making has proof of principle in the monogenic form of diabetes called MODY, which is characterised by defects in the development of the pancreatic islet cells and insulin secretion. The effective stratification of polygenic diabetes (type 1, type 2 and gestational diabetes), while highly appealing, is more challenging though, as complex diabetes manifests through defects in multiple organs, tissues and pathways [40] and is influenced by a wide range of environmental risk factors [41].

The stratification approaches reported to date for polygenic diabetes have used clinical phenotypes (e.g. BMI, C-peptide or HbA_1c_) [42], continuous glucose monitor-derived data [43] or genotypes [44–46] to stratify diabetes into aetiological subclasses. One of the earliest attempts to do this derived three diabetes subtypes by clustering data from electronic medical records and regressed genotype array data against each subtype to provide sets of SNPs from which pathophysiological inferences were made [44]. This approach is prone to type 1 error, owing to the large number of parallel hypothesis tests performed, the liberal significance threshold employed when selecting SNPs and the manner in which biological function was assigned to SNP sets (which may be prone to bias owing to the type of data available at that time). By contrast, the more recent studies using SNP clustering approaches [45, 46] are less prone to bias or type 1 error, as a very conservative *p* value threshold is used when selecting SNPs and the pathogenicity of variants is determined through very large and well-phenotyped independent datasets. Key barriers to the clinical translation of these approaches is that most use probabilistic soft clustering methods, which do not classify most individuals into discrete subtypes of diabetes, but instead assign one or more probabilities linking the individual to one or more subtypes of diabetes.

Udler et al derived process-specific clusters using enhancer enrichment from multi-cell epigenomic data [46], which were used to inform the design of polygenic risk scores (PRS), where higher scores were associated with relevant clinical outcomes (e.g. hypertension, coronary artery disease and stroke). These process-specific clusters characterised: (1) elevated beta cell function, (2) diminished beta cell function, (3) insulin resistance, (4) lipodystrophy-like adipose distribution and (5) disrupted liver lipid metabolism. Mahajan et al described similar clusters [45], but did not proceed to link these with clinical traits through participant-level association analyses*.* Thus, using these approaches to re-diagnose an individual with a new subtype of diabetes in a clinically meaningful and actionable manner is challenging.

Overall, these studies have provided intriguing insights into the aetiology of diabetes and helped to elucidate the factors that drive disease progression. However, many of these clustering methods do not assign most individuals to distinct clusters or risk misclassifying people to incorrect diagnostic categories (because most people are not defined by a distinct subtype of diabetes). Those methods that do not seek to assign individuals to distinct clusters focus on assigning probabilities of belonging to one or more clusters, which may be difficult to utilise in current clinical settings and may be less powerful than algorithms using continuous data [47].

Richardson et al [48] have categorised the existing omics integration approaches into vertical and horizontal methods. Vertical integration can be viewed as the combination of multiple ‘layers’ of data usually derived from the same individual. By contrast, horizontal data integration incorporates the same type of data derived from separate cohorts. Ritchie et al [49] describe multi-staged analyses, where associations are tested within data types (i.e. SNP datasets), filtered and then tested against traits, with the limitation of assuming linearity; meta-dimensional analysis simultaneously integrates various data types into a single model.

In a step towards clinical translation of omics data, a recent analysis assigned the participants whole-genome sequences pathogenicity scores according to the American College of Medical Genetics guidelines, revealing that one in every six participants carried at least one pathogenic variant [50]. Clinical biochemistry, metabolomic and digital imaging data (from MRI, CT, ECG, echocardiography, continuous glucose monitoring), as well as information from the participant’s medical records and family history were subsequently combined to derive a set of clinically relevant phenotypes relating mainly to cardiac and endocrine disorders (including type 2 and syndromic forms of diabetes). Associations were then tested between pathogenic variants and these clinical phenotypes, revealing that one in nine participants carried pathogenic variants that mapped to relevant clinical traits [50]. These findings imply that the appropriate use of deep-phenotyping data may enhance the ability to discriminate between high- and low-risk individuals with conventional risk factors and/or disease characteristics.

## Summary and conclusions

The major expansion of accessible omics data in large epidemiological cohorts provides unprecedented opportunities for diabetes research and practice. Breakthroughs in knowledge will require training in analytical methods to keep pace with data generation; with very large and complex datasets, tasks that were once considered simple, such as data handling and quality control, now often require specialist training. The analyses that follow, possibly focusing on casual inference, gene–environment interactions, pharmacogenomics or functional annotation, will require other types of specialist knowledge. Many of these analyses will make use of external datasets that help establish biologically meaningful connections between molecular phenotypes, which requires specialist knowledge to access, integrate and interpret this information. Thus, appropriate training in specialist analytical tasks will be increasingly important for the next generation of epidemiologists. Importantly though, this should be balanced against the need for education in the core tenets of epidemiology, so that conclusions drawn from complex analyses are accurate and meaningful.

## Electronic supplementary material

Figure slide (PPTX 162 kb)

## Abbreviations

EPIC: European Prospective Investigation into Cancer and Nutrition
FDA: Food and Drug Administration
GWAS: Genome-wide association studies
IMI: Innovative Medicines Initiative
MR: Mendelian randomisation
