# Supplementary material for: Next-generation epidemiology: the role of high-resolution molecular phenotyping in diabetes research
Source: Diabetologia. 2020 Aug 25;63(12):2521–32. doi: 10.1007/s00125-020-05246-w (PMC7641957; doi:10.1007/s00125-020-05246-w)
Supplement: Supplementary file 1 — (PPTX 162 kb) [file 125_2020_5246_MOESM1_ESM.pptx]

## Slide 1
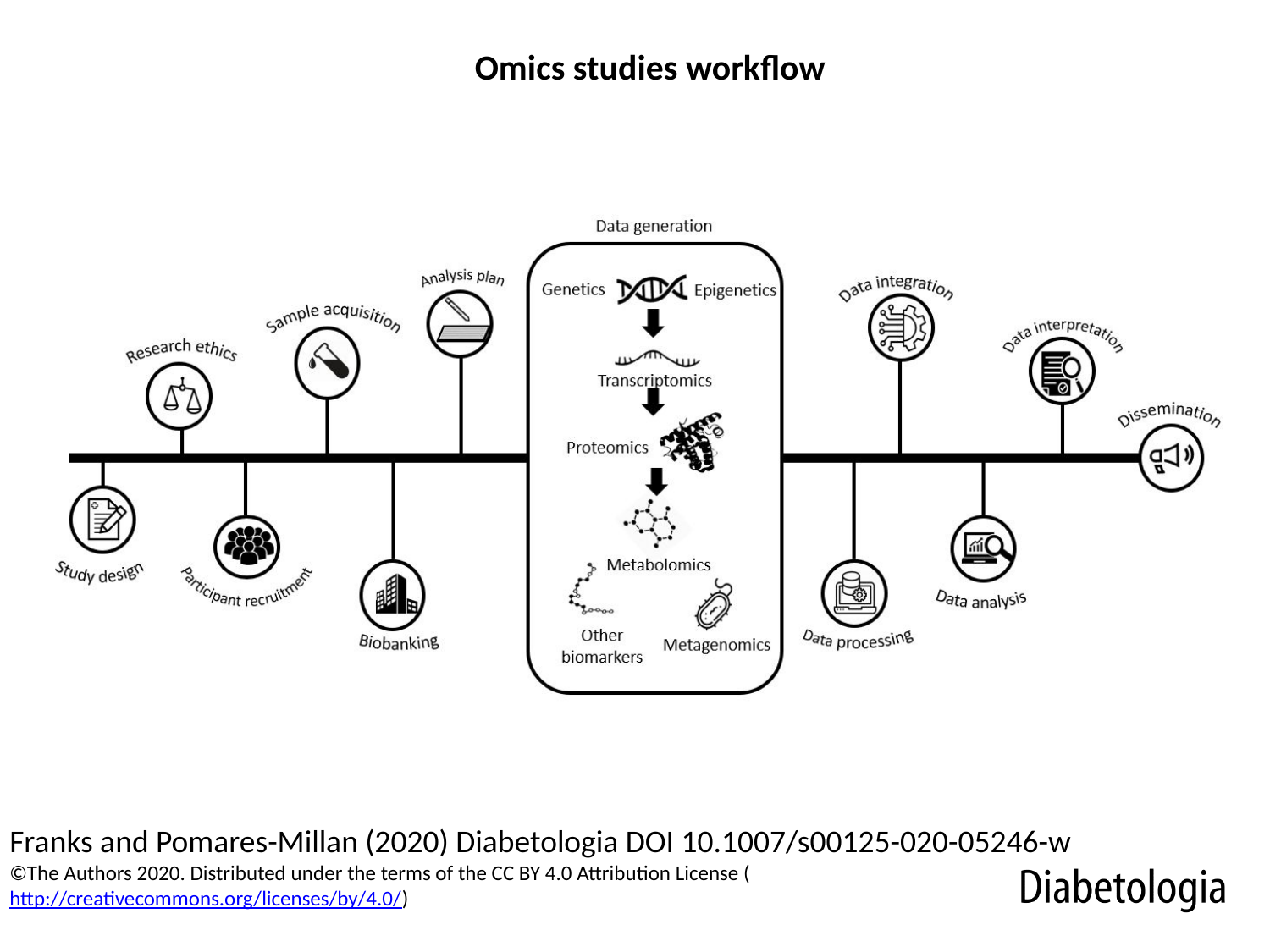

Omics studies workflow
Franks and Pomares-Millan (2020) Diabetologia DOI 10.1007/s00125-020-05246-w
©The Authors 2020. Distributed under the terms of the CC BY 4.0 Attribution License (http://creativecommons.org/licenses/by/4.0/)
